# Utilitarianism and the pandemic

**DOI:** 10.1111/bioe.12771

**Published:** 2020-06-11

**Authors:** Julian Savulescu, Ingmar Persson, Dominic Wilkinson

**Affiliations:** ^1^ Oxford Uehiro Centre for Practical Ethics University of Oxford Oxford United Kingdom of Great Britain and Northern Ireland; ^2^ Wellcome Centre for Ethics and Humanities University of Oxford Oxford United Kingdom of Great Britain and Northern Ireland; ^3^ Visiting Professorial Fellow in Biomedical Ethics, Biomedical Ethics Research Group Murdoch Children’s Research Institute Melbourne Australia; ^4^ Distinguished Visiting Professor in Law, Melbourne Law School University of Melbourne Melbourne Victoria Australia; ^5^ Department of Philosophy, Linguistics and Theory of Science Gothenburg University Gothenburg Sweden; ^6^ John Radcliffe Hospital Oxford United Kingdom of Great Britain and Northern Ireland

**Keywords:** COVID‐19, pandemic ethics, resource allocation, utilitarianism

## Abstract

There are no egalitarians in a pandemic. The scale of the challenge for health systems and public policy means that there is an ineluctable need to prioritize the needs of the many. It is impossible to treat all citizens equally, and a failure to carefully consider the consequences of actions could lead to massive preventable loss of life. In a pandemic there is a strong ethical need to consider how to do most good overall. Utilitarianism is an influential moral theory that states that the right action is the action that is expected to produce the greatest good. It offers clear operationalizable principles. In this paper we provide a summary of how utilitarianism could inform two challenging questions that have been important in the early phase of the pandemic: (a) Triage: which patients should receive access to a ventilator if there is overwhelming demand outstripping supply? (b) Lockdown: how should countries decide when to implement stringent social restrictions, balancing preventing deaths from COVID‐19 with causing deaths and reductions in well‐being from other causes? Our aim is not to argue that utilitarianism is the only relevant ethical theory, or in favour of a purely utilitarian approach. However, clearly considering which options will do the most good overall will help societies identify and consider the necessary cost of other values. Societies may choose either to embrace or not to embrace the utilitarian course, but with a clear understanding of the values involved and the price they are willing to pay.

## 1 INTRODUCTION

The COVID‐19 pandemic has posed a formidable and virtually unprecedented challenge to health professionals, health systems and to national governments. The potential threat to large numbers of patients has led to restrictions on movement, employment, and everyday life that have impacted the lives of billions and come at massive economic cost. Health systems, facing existing or predicted demand overwhelming capacity, have generated guidelines indicating which patients should receive treatment.

One ethical theory has been both cited and criticized in public debate about pandemic response.

The civil rights office of the US Department of Health and Human Services stated that:

persons with disabilities, with limited English skills, or needing religious accommodations should not be put at the end of the line for health services during emergencies. Our civil rights laws protect the equal dignity of every human life from ruthless utilitarianism.^1^

After the *New York Times* reported that some state pandemic plans instructed hospitals not to offer mechanical ventilation to people above a certain age or with particular health conditions (e.g. ‘severe or profound mental retardation’ as well as ‘moderate to severe dementia’), the Office for Civil Rights (OCR) responded: ‘… persons with disabilities should not be denied medical care on the basis of stereotypes, assessments of quality of life, or judgments about a person’s relative “worth” based on the presence or absence of disabilities or age’.^2^

Utilitarianism is now often used as a pejorative term, meaning something like ‘using a person as a means to an end’, or even worse, akin to some kind of ethical dystopia.^3^ Yet utilitarianism was originally conceived as a progressive liberating theory where everyone’s well‐being counted equally. This was a powerful and radical political theory in the 19th century, when large sections of the population were completely disenfranchised and suffered from institutional discrimination. The theory played a role in antislavery, women’s liberation and animal rights movements. Yet utilitarianism remains relevant in the 21st century. As we will discuss, it may be particularly salient and important to consider in the face of global threats to health and well‐being.

In this paper, we will summarize what utilitarianism is and how it would apply to the COVID‐19 pandemic. Our aim is not to argue that utilitarianism is the *only* relevant ethical theory, or that a *purely* utilitarian approach must be adopted. However, it is important to note that whenever a utilitarian solution to a dilemma is adopted, there will be more well‐being or happiness in the world. Typically, some people will be better off. Of course, there may be good ethical reasons to deviate from a pure utilitarian approach, for example in order to protect rights or promote equality. However, considering the alternative will help societies to identify and consider the necessary cost of these other ethical values. Utilitarianism is not the end of ethical reflection, but it is a good place to start.

### 1.1 What is utilitarianism?

Most moral theories imply that there is a (moral) reason to do what is expected to maximize what is good for all, or more precisely, the net surplus of what is good for all over what is bad for them. This might be called *a principle of beneficence*. Utilitarians hold that maximizing what is good for all is all there is to morality. It makes moral decisions simple by supplying a single measure of rightness: maximization of utility. In many situations this may be enough, along with rules of thumb with the help of which it could be determined what maximizes utility.

According to most moral theories there are, however, other moral reasons. For instance, utilitarianism has often been criticized for ignoring the question of what is *a just or fair distribution* of what is good for all. The outcome that generates the greatest good overall may be different from the outcome whose distribution of goodness comes closest to being just or fair. Then the principle of beneficence will have to be balanced against the principle of justice. This will most likely have to be done in an intuitive way. It is very controversial what a just or fair distribution consists in, e.g. whether it consists in getting what is deserved or in more equal shares. This is far too controversial to be settled here. It follows that the issue of balancing justice and beneficence against each other must also be left aside.

Another moral principle is a *principle of autonomy,* which gives weight to an individual’s freedom to choose and to determine, for themselves, how to live their own life. Individual freedoms may conflict with overall good, for example, when individuals choose to flout social distancing laws, or when individuals demand a scarce resource for themselves or their family members. This also brings us to the issue of whether the principle of beneficence should be impartial and accord the same moral weight to the good of all other individuals or whether it should allow greater weight to the good of those who are close to us (and to human over non‐human beings). For the purpose of discussing what policies societies should adopt to deal with pandemics, it is reasonable to assume impartiality.

A further issue is what constitutes goodness and badness for individuals. According to the most familiar theory, *hedonism*, what is intrinsically good consists in various positive experiences, of pleasure and happiness. What is intrinsically bad consists in negative experiences of pain and unhappiness. Hedonism is, however, frequently criticized for being too narrow in not recognizing that what we are not aware of can be good or bad for us, e.g. that our partners deceive us, or that the state surveys our behaviour, so cleverly that we never notice it. For such reasons a wider conception of what is intrinsically good or bad for us than hedonism will be assumed here, though to determine its precise import would take us too far afield.

Some moral theories imply that there is a stronger or more stringent moral reason to *omit doing harm* than to *benefit*. Thus, they imply that there is a stronger reason to avoid making things worse for somebody by killing them, causing them injury or pain, than to benefit them by preventing them from being killed, injured, etc. With respect to pandemics, considerable moral weight has been attached to harms such as death and disease that can be prevented by various constraints. Therefore, for the present discussion it is better to proceed on the assumption that there is no significant moral difference between harming and omitting to benefit.

Utilitarianism typically accepts that instances of goodness and badness can *be aggregated in a quantitative fashion*. Thus, consider a very mild pain that is caused by a physical stimulus of one unit and that lasts for 10 min. Now compare 100 instances of such a pain either spread out over 100 lives or over one life lasting many decades with a single instance of excruciating pain caused by 75 units of the physical stimulus lasting for 10 min. According to a standard utilitarian calculus the former outcome is worse than the latter, but this seems implausible. Most of us would prefer 100 instances of mild pain dispersed over our lives than 10 min of excruciating pain. It might be thought that this issue is crucial in the present context, since we will have to balance the deaths of a lower number of people against smaller burdens for a much higher number of people. We will, however, see that what is morally relevant from a utilitarian perspective isn’t death in itself but rather the length and quality of life the deceased would have had if they hadn’t died.

It might be said that what matters in the end is what action *actually* maximizes what is good for all rather than what action *is expected* to maximize what is good for all. But our best guide to what will actually happen is what is expected to happen on the best available evidence. So, when we decide what to do, we have to go by what is predicted to be best. This is true in most situations (although in some special cases we know that what is expected to be best is not what will actually be best^4^). The expected utility of an action is the sum of the products of the probability and value of each of the possible outcomes of that action.

### 1.2 Act and rule utilitarianism

There are two broad schools of utilitarianism. According to act utilitarianism, the right act is the act that produces the best consequences. According to rule utilitarianism, the right rule is the rule that produces the best consequences. The law is often an instantiation of rule utilitarianism: laws are chosen because they bring about the best consequences.

These versions of utilitarianism can come apart. Sometimes an act will clearly have better consequences, or no adverse consequences but a rule proscribes that act.

Principles or laws around non‐discrimination are examples of this. Not considering a person’s advanced age or severe disability (e.g. severe dementia) in the allocation of resources, including ventilators, might mean that another person is unable to access those resources who would have gained greater benefit from it, against act utilitarianism. Yet the rule might still overall have better consequences if the non‐discrimination rule has over‐riding benefits.

### 1.3 Two level utilitarianism

The two different schools of utilitarianism can be combined. The father of modern utilitarianism, Richard Hare, argued that moral thinking occurs at two levels: intuitive and critical, and that we should move between these depending on the circumstances.^5^ At the intuitive level, we have many rough rules of thumb that can be rapidly deployed without protracted and demanding reflection: don't kill, don't steal, be honest, etc. These enable us to act efficiently in everyday life. During a pandemic, doctors and other decision‐makers require rules of thumb. For example, when faced with multiple simultaneous patients in the emergency department it is important to have a way of reaching a decision quickly about which patient to attend to first. Triage rules are potentially justified by a form of rule utilitarianism that enables rapid intuitive decisions.

‘Critical level’ utilitarianism requires choosing the action that will maximize the good when we are thinking in the ‘cool, calm hour’, with all the facts at hand. Hare imagined a decision‐maker who had perfect knowledge of the outcomes of all available options (he called them a ‘utilitarian archangel’). In complex situations, where there is time to do so, we must try to rise to the more reflective and deliberative critical level and ask what action we should endorse. What really is the right answer? Hare argues that in such situations we should employ act utilitarianism (this corresponds to system 1 and 2 thinking in psychology^6^).

We will explore some of the implications of critical level utilitarianism for the current COVID‐19 pandemic. We will also describe plausible rules of thumb that would tend to maximize utility and would be useful in emergency and urgent situations. Box 1 illustrates two questions that have been prominent in the early phase of this pandemic.

Box 1 1 *Alessandro is a 68‐year‐old doctor. He has moderate chronic obstructive airways disease. He contracts COVID‐19 while caring for patients with the same disease. He develops respiratory failure. Jason is a 52‐year‐old businessman who contracted COVID‐19 while travelling for business reasons. He is otherwise well but develops respiratory failure*.

The triage question: There is only one ventilator remaining. Who should receive ventilation?

*The UK government received modelling that predicted that COVID‐19 would lead to 500,000 deaths in the absence of measures to reduce spread. This could be reduced to 20,000 by implementing major social distancing measures (lockdown). The economic effects arising from restriction of liberty will predictably result in large numbers of job losses, mental illness, and increased medical risk (e.g. unemployment is associated with increased risk of coronary heart disease)*. 7 *Cancellation of elective operations and interventions will result in prolongation of suffering and potentially death. Those suffering from non‐COVID illness may not be able to receive treatment in hospital because there are no beds available*.

The lockdown question: How should we balance preventing deaths from COVID‐19 with causing deaths and reductions in well‐being from other causes?

### 1.4 Utilitarian rules of thumb

There are several rules of thumb that can guide rapid decision‐making about these kinds of cases.

**1. Number**

One utilitarian rule of thumb is to save the greatest number (other things being equal). This rule could be applied to the lockdown question by assessing how many lives would be lost if lockdown is applied, or not applied. It could also be used for the triage question: in practice, this would mean considering the following variables:

A. Probability

If Jason has a 90% chance of recovery and Alessandro has a 10% chance, other things being equal, you should use your ventilator for Jason. Indeed, if you treat people like Jason rather than people like Alessandro, you will save nine people instead of one for every 10 treated. That is why probability is a relevant consideration.^8^

B. Duration of treatment

In a setting of scarcity, duration of time on a ventilator has implications for the numbers of lives saved. The longer one person will be on a ventilator, the more people who potentially die because they cannot get access to breathing support. If Alessandro needs a ventilator for 4 weeks, and four others (including Jason) need it for 1 week, the choice is between saving one person or four people. So doctors should take duration of use into account.

C. Resources

When resources are limited, resources equate to numbers of lives. The more resources a treatment or a person uses, the fewer are available for others. Imagine that Alessandro and Jason had identical chances of survival, but Alessandro needed a treatment that required three staff to administer the treatment (e.g. extracorporeal membrane oxygenation [ECMO]—essentially cardiac bypass) and Jason needed a treatment that required only one staff member (e.g. mechanical ventilation). We can potentially save three people with ventilation for every patient we save with ECMO. ECMO should be a lower priority than ventilation.

**2. Length of life**

According to utilitarianism, how long a benefit will be enjoyed matters—it affects the amount of good produced. Thus for life‐saving treatment, treatment that saves people’s lives for longer is to be preferred over treatments that save life for shorter periods.

According to this criterion, priority should be given to the younger Jason rather than the older Alessandro, because Alessandro is expected to live less long if successfully treated. If it were Jason who was expected to die sooner, utilitarianism would support treating Alessandro, even though he is older.

Age is thus a de facto measure of length. Because older people tend to die sooner than younger people, utilitarianism tends to favour saving the lives of the younger. However, age itself does not matter: it is the expected length of the benefit. This is why utilitarianism is not unfairly discriminatory, and not ‘ageist’ in an ethically problematic sense (we will discuss discrimination further below).

**FIGURE 1:**
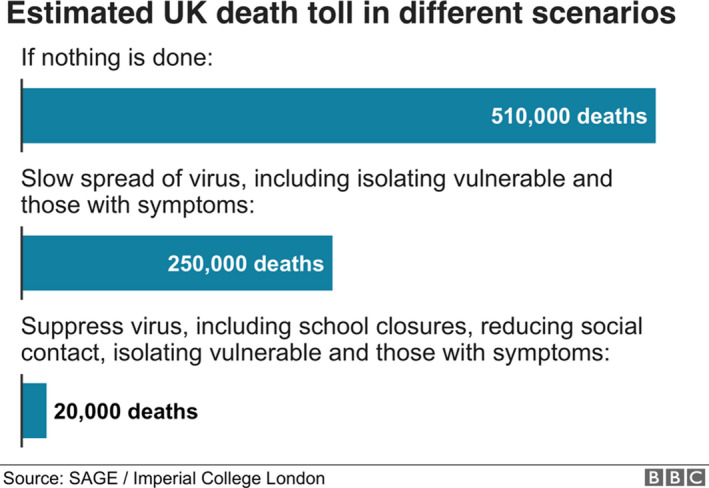
Estimated UK death toll in different scenarios. Figure retrieved from https://www.bbc.co.uk/news/health‐51979654 but no longer available^46^

Length of life is also relevant for the lockdown question. It is the length of life extended that matters. This has implications for evaluation of current policy. In the UK, the decision to implement national lockdown at the end of March was influenced by modelling produced by Imperial College (Figure 1).

The UK Government opted to try to reduce deaths to 20,000. But it was not clear from the modelling figure of 500,000 how many of these people would have died anyway from other causes,^9^ or relatively soon after not contracting COVID‐19. Every year more than 600,000 people die in the UK. For utilitarians, the number of lives saved is irrelevant—it is how long these lives would be prolonged by the intervention.

The average age of death of COVID‐19 patients in Italy was 78.^10^ This implies that many of those saved by implementing lockdown would have short life expectancies. The average life expectancy at age 80 is 9 years, and overall, COVID‐19 has been estimated to lead to a loss of 11 life years on average. According to utilitarianism, the value of a year of full quality life is the same regardless of how old a patient is. However, if the pandemic largely affects patients with short life expectancy, the benefit of a lockdown (preventing deaths) would be smaller than a different illness that affected younger patients. The cost of lockdown per year of life saved could be astronomical, when one considers all costs including economic and wider social effects.

At the end of March, economists van den Broek‐Altenburg and Atherly, from the University of Vermont estimated the cost‐effectiveness of implanting large scale protective measures to reduce the spread of COVID‐19. They calculated the cost per Quality Adjusted Life Year (QALY) of a $US 1 trillion economic stimulus package against the number of lost life years potentially averted (up to 13 million in the USA). They estimated that such a package would cost between $75,000–650,000 per QALY.^11^ (The US government subsequently approved a $US 2 trillion stimulus package.) That suggests that such measures are unlikely to be cost‐effective according to the usual thresholds applied to the costs of medical interventions to save lives. For example, the upper limit for cost‐effectiveness of an intervention in the USA is often taken to be about $100,000 per year of life saved.^12^

There are two points to make about such an analysis. The first is that assessing the utilitarian answer to the lockdown question is highly dependent on the specific factual answers—the *harm averted by* acting, the *harm caused by* acting. It is exceedingly difficult to determine which course of action would be best from the point of view of critical level utilitarianism, partly because of enormous uncertainty about the relevant facts. Secondly, even if lockdown were cost‐effective, it would not be as cost‐effective as different interventions that save babies or young people. For example, if an intervention saved the life of a younger person with a different disease for 50 years, you would only have to save one‐fifth as many to bring about as much benefit. It costs a few dollars to save the life of a child in a developing country.

While interventions to prevent COVID‐19 may be cost‐effective (though this seems perhaps unlikely), they are unlikely to be the *most* cost‐effective actions that we could take. There are likely to be better investments for utilitarians. As an example, The Gates Foundation has estimated that global eradication of malaria by the year 2040 would cost up to $120 billion.^13^ Such an initiative (costing only 1/15th as much as the US pandemic stimulus package)^14^ would potentially save 11 million lives.

**3. Quality of life**

Utilitarians consider not just how long someone will live after treatment but how well they will live. They consider quality of life important.

This could be relevant to the triage question (as suggested in the quote from the Office for Civil Rights at the start of this paper).

Consider an extreme example. The end point of dementia is unconsciousness. Imagine that of our two patients with respiratory failure Alessandro is still working, in possession of all of his faculties. Jason, by contrast (in this version of the case) has end stage dementia. According to utilitarians, we should treat Alessandro if we cannot treat both. Jason would derive zero benefit from being kept alive in an unconscious state. Indeed, this would apply potentially even if Jason (with dementia) had a higher chance of survival, or were going to survive for longer.

What about lesser degrees of cognitive impairment or other disabilities? According to utilitarians, these would also be considered in making allocation decisions if they affect the person’s well‐being.

However, comparisons of overall well‐being between individuals are not straightforward. It is not necessarily the case that someone with a disability would have lower well‐being than someone without a disability. Probably the most profound question in ethics is what makes a person’s life good, or constitutes well‐being. Philosophers have debated this question for thousands of years. Answers include happiness, desire fulfilment or flourishing as human animals (which includes having deep relationships with others and being autonomous, amongst other things).^15^

As a heuristic for triage, it may be that in developed countries a threshold is set at a level where overall well‐being is certain to be low.^16^ One practical cut off would be unconsciousness or severe disorders of consciousness, such as being in a minimally conscious state. It is highly unlikely to be cost‐effective to provide intensive care for a patient who is permanently minimally conscious.^17^ Lines could be drawn where there is more uncertainty, and may need to be in countries with more limited resources, or if the demand were much greater. For example, the threshold might be set at the ability to recognize and respond meaningfully with other people. So, on this approach, cognitive impairments that reduced the capacity to have minimal human relationships would reduce priority for treatment as a proxy for believed reduced well‐being.^18^

Quality of life may also be relevant to the lockdown question. If the life years saved by lockdown were likely to be of reduced quality that would influence how much benefit overall is gained, and therefore what economic cost would be worth incurring.

**4. Equivalence of acts and omissions, withdrawing and withholding**

For utilitarians, how an outcome arises is morally irrelevant. It makes no difference if it is the result of an act, or an omission.

Doctors, patients and families, however, hold that there is a moral difference between acts and omissions. Many people hold a causal account of responsibility: they tend to think that we are responsible for the consequences of our acts but not for our omissions. Thus people tend to believe that withdrawal of life‐sustaining treatment is morally worse than withholding life‐sustaining treatment.

This folk commitment to a causal sense of responsibility and the acts/omission distinction has a number of bad consequences.

It means that there is considerable attention in pandemic guidelines to decisions about initiation of treatment. The ‘triage question’ is largely or entirely focused on whether to start treatment. Withholding of treatment from patients with poorer prognosis is often thought to be ethically acceptable. However, some apparently poor prognosis patients will do well and a trial of treatment might provide more accurate prognostic information. Thus, under conditions of uncertainty, a trial of treatment with the possibility of withdrawal would be preferable to withholding treatment.^19^

Utilitarianism would reject the idea of employing any form of ‘first come, first served’ to decide about treatment. The timing of when a patient arrives needing treatment is morally irrelevant to whether or not they should receive treatment. This is a principle that we have elsewhere labelled the *principle of temporal neutrality*. According to utilitarianism, doctors should be prepared to withdraw treatment from poor prognosis patients in order to enable the treatment of better prognosis patients if they arrive later.

Consideration of acts and omissions is also relevant to wider social questions raised by the pandemic. Failing to implement a good policy is equivalent to actively implementing a bad policy, when the outcome of the two decisions is the same. So utilitarians hold policy makers responsible not only for what they do, but for what they fail to do. Failing to implement other policies, with the result of avoidable, foreseeable deaths is equivalent to killing for utilitarians. (This means that policy makers are just as blameworthy for failing to eradicate malaria as they would have been if they had failed to act in response to coronavirus.)

**5. Social benefit**

According to utilitarianism, all the consequences of actions, both short and long term, direct and indirect are relevant to decisions. Thus it may be relevant to consider not only the benefit to the person directly affected by an action (for example, by being placed on a ventilator), but also others. This can be called ‘social benefit’ or social worth.

In pandemics, one rule of thumb likely to maximize utility would be to give priority to health care workers, those providing key services and others who are necessary to provide essential benefits to others. This has been applied in many countries, including the UK, to testing for coronavirus. However, it might also apply to access to ventilators or other medical treatments. A reason given for this is that it will potentially mean that they can also return to work sooner.^20^

What about the social worth of others? Should criminals have a lower priority in accessing limited resources? What about scientists working on a vaccine? Related to social benefits is the issue of dependents. Should pregnant women and parents of dependent children be given greater priority for health care? Developing rules of thumb for assessing social worth is ethically and epistemically complex, liable to abuse and difficult to enforce fairly. Critical level utilitarianism would likely not endorse such priority rules, perhaps beyond prioritizing critical essential services workers (which is relatively clear cut and easy to enforce and has wide social acceptance).

Utilitarianism is sensitive to the potential for abuse of its operationalized principles. If there is a risk that a principle will be abused, this should be taken into account in deciding whether to operationalize it or not. For example, social worth is easily abused by the powerful to claim privilege and priority.

**6. Responsibility**

For utilitarians, we are morally responsible to the extent that the effects of our acts or omissions are foreseeable and we have control over them. Intentions are irrelevant for utilitarians. It is not what we want to happen that matters: it is what we can foresee, and what actually happens. So even if consequences are unintended, we are still responsible if they are foreseeable and avoidable.

This implies that *failing to take* a course of action that would bring about more good, or avert more harm, is equivalent to *intentionally causing* that harm. The moral responsibility for choosing an inferior policy is high for utilitarians and actions that result from this are subsequently blameworthy.

Utilitarianism is a very demanding theory in several ways. Whenever we foreseeably and avoidably bring about a less good state of affairs, we are morally responsible and blameworthy. If bringing about the best policy requires more research, we are responsible for the deaths that occur because that research was not done.

Another issue in resource allocation is responsibility for illness. Many people have the intuition that responsibility for illness should be taken into account in the allocation of limited resources. Smokers should receive lower priority for lung transplants, drinkers for liver transplants. The UK government has also encouraged the public to take responsibility for their health.^21^ In the case of COVID‐19, people with various comorbidities have worse prognoses. For example, type II diabetes is one such comorbidity, and its risk factors include so‐called 'lifestyle' factors such as diet and exercise.

There are numerous problems with trying to use responsibility for illness in the allocation of resources.^22^ Utilitarians eschew all direct consideration of causal contribution to illness and, indeed, any ‘backward looking’ considerations like desert. They are only concerned with bringing about the best outcome. If, for example, diabetes reduces the chance of survival, it is relevant insofar as it reduces the chance of survival, not because it was the result of any voluntary behaviour.

Responsibility (or the disposition to behaviour that led to ill health) is only relevant for utilitarians insofar as it affects probability, length or quality of survival. This is in line with how responsibility is generally used in the NHS.^23^

**7. Avoid psychological biases, intuitions and heuristics**

Utilitarianism seeks to avoid biases, emotions, intuitions or heuristics that prevent the most good being realized.

For example, humans are insensitive or numb to large numbers.^24^ They are also more moved by a single identifiable individual suffering than by large numbers of anonymous individuals suffering each to the same extent (this is the so‐called ‘rule of rescue’^25^). Thus they will be motivated to alleviate the suffering of a single highly publicized individual, rather than taking action that prevents suffering of a larger amount of unknown or unidentifiable individuals. To some extent, national responses to COVID‐19 might represent a massive form of the ‘rule of rescue’.

Probably most relevant to political decision‐making is bias towards the near future. The desire to avoid deaths now is stronger than the desire to avoid deaths in the future. It is psychologically easier to impose severe lockdown now in the name of saving lives threatened now, even if the toll of loss of life would be greater in the future. There is some evidence that the lockdown and related factors such as reduced access to medical care are leading to additional deaths from causes other than coronavirus.^26^ It might be anticipated that there will be large numbers of future deaths caused by the economic downturn induced by the pandemic. After the 2008 financial crash it is estimated that there were 250,000 excess cancer deaths just in Organisation for Economic Co‐operation and Development countries.

These future and non‐identifiable deaths might be greater than or less than those prevented by lockdown. They are hard to predict and even to confidently assign, which is one reason that they are difficult to take into account. However, they are just as ethically relevant as the deaths caused by COVID‐19. We should not ignore them because they are less psychologically real and motivating.

Utilitarianism aims to the maximize the good, impartially conceived. Statistical lives matter as much as identifiable lives.

Another bias is to one’s family and friends. According to utilitarianism, we should give equal weight to the lives of strangers, even those in other countries. The effects on the pandemic in Africa are yet to be documented or manifest. Given that there are fewer advanced life support systems, the mortality is likely to be greater. Utilitarianism would favour diverting resources there if the effects would be greater.

Much of ordinary decision‐making is driven by emotion, biases and heuristics. Thus, much of utilitarianism will strike ordinary people as counterintuitive.

### 1.5 The triage question

**FIGURE 2:**
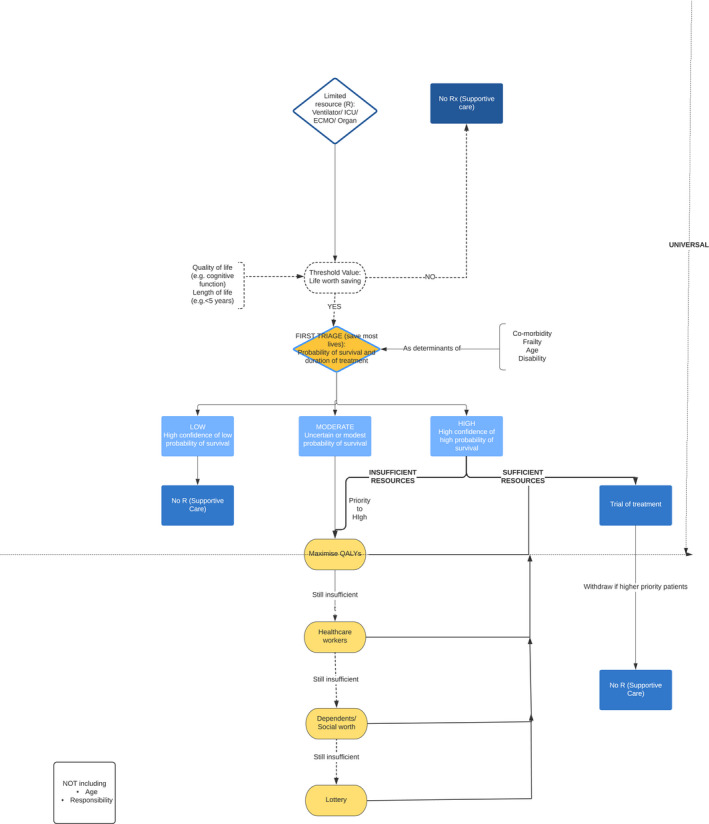
An ethical algorithm for rationing life sustaining treatment

The above rules of thumb could be assembled into an algorithm for allocation of ventilators (Figure 2). Such an algorithm could be used to inform rapid decisions if there were overwhelming numbers of patients presenting in future surges relating to COVID‐19. Alternatively, it might be used to inform decisions about highly scarce and expensive treatments such as ECMO. Because of the need for rapid decisions, based on limited information, this represents an attempt to guide ‘intuitive level’ decisions in a way that would generate most benefit overall. It is thus different from what act utilitarianism (or the critical level approach) would recommend.

The algorithm divides decision‐making into stages, and prioritizes on the basis of different criteria, depending on the availability of resources. For example, it starts by giving highest priority to those with the highest chance of surviving and needing the lowest duration of treatment. This would maximize the number of lives saved. If there are sufficient ventilators to treat all patients with at least a moderate chance of surviving, there would be no need to invoke other criteria. Thus, for example, health care systems with ample pre‐existing intensive care capacity, or who have been able to expand their capacity acutely, might have no need to ration on the basis of life expectancy or quality of life.

If there are insufficient ventilators, additional principles might be invoked. As noted, utilitarianism does not necessarily seek to save most lives, but would aim to achieve the most well‐being overall, including elements of both length of life and quality of life. At a second level, triage might assess both of these factors for patients in need of treatment. In practice, however, estimation of predicted quality adjusted life years for individual patients is highly complex (and may be uncertain). It would be quicker to set a threshold of length and quality of life worth saving. As an example, we have suggested that a health system under severe pressure might elect to only provide mechanical ventilation to patients predicted to survive for at least 5 years with normal quality of life, but the specific threshold used will depend on the level of resource availability and on the level of demand.

### 1.6 The lockdown question

While the triage question lends itself to heuristics, and the development of a rule that might generate the best outcome overall, it is difficult to know what intuitive‐level response would be best for the lockdown question. Because of the scale of the impact of the pandemic, there is a danger that rapid rule‐based responses might go badly wrong and lead to a much worse outcome overall. Instead, this is a question that would be better answered by drawing on critical level utilitarianism. In large part because of uncertainty, there are different views about which strategy for entering or leaving lockdown would generate the best outcome overall. For example, there remains debate about whether the approach in Sweden (avoiding a national lockdown) is better or worse than the approach of Sweden’s Scandinavian neighbour Norway, which implemented a lockdown in early March. At the time of writing, Sweden has reported 2,769 deaths, (274 deaths/million population), compared with 214 deaths in Norway (39/million population).^27^

The important issue for utilitarians is not the number of deaths, but the QALYs lost. Because a large proportion of the deaths in Sweden are in care homes, there may be fewer QALYs lost than a policy that caused a smaller number of avoidable deaths of younger, healthier people. What is important is whether the QALYs lost in Sweden are greater or less than Norway, overall, as a result of the policy. It is far from clear at this point the answer to that question.

Moreover, there can be difficulties in comparing countries, since they differ in more than just the policy applied. They may also differ in other characteristics. The mortality of Stockholm stands out in Sweden: half of Sweden's deaths were in Stockholm, yet its population is roughly 1/5^th^ of Sweden’s: specifically, 1,428 out of 2,854 deaths (May 5, 2020). The mortality rate of a region in the south of Sweden with a population of 1.4 million was half that of Oslo, the capital region of Norway (April 21, 2020), in spite of not having had a lockdown policy for 5 or 6 weeks. The number of deaths in this southern region is 78 compared to 1,428 in Stockholm whose population is only a couple of hundred thousand greater (May 5, 2020). One potential explanation for differences in mortality relates to differences in population density. Another relates to the amount of circulating coronavirus prior to any change in community behaviour (which may or may not have been imposed formally as a lockdown). A further factor may be whether the virus has had access to vulnerable groups. The virus may have been more effectively kept out of aged care in the south of Sweden. That it isn’t simply due to a national lockdown is confirmed by the fact that this mortality figure is lower both than that of the neighbouring Danish capital, Copenhagen, 293, and the county surrounding it, 93 (May 5, 2020), despite that fact that shops, etc. have been locked down in Copenhagen since mid‐March.

It might be that conditions all over Sweden will soon be worse than in Norway and Denmark because of the absence of a national lockdown. However, it is possible that Norway and Denmark’s approach might lead to more deaths at a later stage because of further surges of the virus when lockdown is relaxed. More importantly, as we have argued, the number of deaths from COVID‐19 at a given point in time is not decisive. The question is which strategy will prevent the most deaths from *any cause* (and more importantly preserve the most years of life in full heath). We must keep in mind the prospect of wider harms to the community as a result of lockdown and the economic consequences.

It is difficult to know what overall strategy would be best. There are several clear points, though about how utilitarianism would inform a policy response to the lockdown question.

### 1.7 Evidence sensitivity

Utilitarianism is highly dependent on accurate information about the world. It requires good evidence. Without good evidence, it is less likely that we would choose means that will bring about the most good.

Utilitarianism is thus complementary to science—it requires science. Thus utilitarianism will urge more research to get better estimates of consequences and probabilities from a wide range of possible courses of action. Utilitarianism invites scientific inquiry. The Swedish approach to lockdown has been informed by epidemiological models of the impact of coronavirus that were lower and less dramatic than some of the models used elsewhere (for example in the UK).^28^ Any modelling or data that is used to inform decision‐making should be openly available and subject to peer review. If the evidence changes, or the modelling needs to be revised, policy should also change. This means that countries might need to change their policy. That could mean relaxing lockdown, or implementing stricter lockdown. The UK government changed tack in its response to coronavirus in late March in response to revised modelling.^29^ That does necessarily mean that the previous policy was mistaken. As noted, utilitarianism directs decisions on the basis of expected utility. Where our expectations change, decisions should change too.

For example, in order to get better estimates of true mortality, utilitarianism would support random population testing to see the incidence of COVID‐19 in asymptomatic or minimally symptomatic community members.

Sometimes the opportunity costs of gathering more information or evidence will be prohibitive when urgent action is needed. In these cases, it is important that beliefs are as rational as possible. They should result from wide expert dialogue, embracing the possibility of dissensus.^30^

### 1.8 Global, impartial equality

Critical level utilitarianism requires impartial and equal consideration of the well‐being of all sentient creatures. In this case, it requires consideration of people now and in the future, as well as people without coronavirus who might be affected by lockdown. It includes the well‐being of all people, old and young, sick and well, in one’s own country and internationally.

This means that it is critical to assess both the well‐being costs of COVID‐19, and the well‐being costs of lockdown. There is currently huge attention to quantifying the numbers of cases of COVID‐19 infection and the number of consequent deaths. However, there is much less attention to the possible consequences of lockdown measures for people without coronavirus. Recent figures (at the end of April) suggest that the UK has had a large increase in all‐cause mortality—the highest in Europe, and that this rate has not been decreasing even as reported deaths from COVID‐19 have fallen.^31^ There is an urgent need to identify and quantify deaths (and more importantly loss of years of well‐being) from all causes in order to inform decisions. Deaths or illness from COVID‐19 might be greater in number than other causes (or they might not), but they are not ethically more important than those from other causes.

Lockdown measures themselves will have direct morbidity and mortality (through denial or delay of medical treatment), as well as indirect effects through economic recession. One estimate is that 25 million jobs will be lost worldwide^32^ with associated loss of well‐being and death.

According to utilitarianism, the right policy is the one that maximizes well‐being overall, across all people across all countries. Utilitarianism embraces radical impartial equality—all well‐being and deaths are equal (other things being equal). The cause of loss of well‐being does not matter. Thus, a utilitarian policy will only invest in preventing loss of life from COVID‐19 provided it is the most efficient way of saving all lives.

We have noted already that other global health priorities might be considerably more cost‐effective than the financial costs of responding to coronavirus. However, there are other important global considerations. The UK has banned the sale of 80 drugs to other countries in a bid to prevent NHS shortages.^33^ From a utilitarian perspective, this may be the wrong course of action if the sale of the drugs would save more lives globally if exported. There may be a moral obligation to help others that overrides the obligation to one’s own citizens. Many countries have sourced large numbers of ventilators in order to be able to meet anticipated demand in their own country. However, the consequences of the pandemic may be much more severe in low and middle income countries (LMIC). Some of the investment that countries have made into their own (already well‐resourced) health care systems would yield much greater benefit for LMIC. That might include making ventilators available (poor countries have been outbid by wealthy countries in the scramble to purchase ventilators).^34^ It might include support for LMIC policies that are less costly but potentially effective ways of averting the crisis (for example, Vietnam employed mass testing and contact tracing to prevent the spread of COVID‐19, and as a result, reported zero COVID‐19 deaths at the end of April^35^). Policy makers in LMIC may benefit from some of the modelling and scientific expertise available in other countries to support their decision‐making. It has been questioned whether isolation will work in Africa or whether it will kill more young people through its economic effects and subsequent malnutrition.^36^

For utilitarians, policy will need to be sensitive to context and facts about individuals and local communities. The policy that is best for one country may be worst for another.

Utilitarianism is a theory with no national boundaries.

### 1.9 Well‐being matters more than rights and liberty

For utilitarianism, well‐being is all that matters. Liberty and rights are only important insofar as they secure well‐being. Thus a utilitarian approach to the lockdown question may be prepared to override the right to privacy or liberty to protect well‐being.

Vietnam, Singapore, Taiwan and China have used methods such as tracing contacts and enforcing self‐isolation using mobile phone data, with severe penalties for failure to comply (in Singapore, it is up to 6 months gaol).^37^ These countries have been highly effective at containing COVID‐19, more so than liberal Western countries with greater emphasis on rights and liberties. Utilitarians support the East Asian approach of constraining liberty and privacy to promote security and well‐being. This approach also appears cost‐effective while delayed response may not be.

One recent suggestion has been an app that facilitates contract tracing.^38^ However, participation in the programme is meant to be voluntary: people would need to agree to share information about their whereabouts and health status. Utilitarianism would favour a more coercive approach if this is more effective. Those who favour such voluntary programmes give greater weight to consent and privacy than to well‐being and life. This is a value choice: it chooses individual rights over overall reduction in the spread of disease. Of course, countries are free to pursue individual freedom, but if the liberty based approach is less effective, it will necessarily come at the cost of additional cases of COVID‐19 and additional deaths.

Importantly, the extent of the liberty restriction or rights violation should be commensurate with the effect on well‐being. Utilitarianism would support isolating certain groups if the benefit to them was greater or the benefit to others was greater. Thus a utilitarian approach to lockdown might favour selective isolation of the elderly and other vulnerable groups if that was the most cost‐effective way to secure overall well‐being.

Likewise, the restriction of liberty of low risk groups may also be necessary to secure large collective benefits. This justifies, for example, in the case of influenza, vaccinating children, who are at low risk of flu complications, in order to protect the elderly, who have less effective immune responses to vaccination and are at greater risk of flu complications.^39^ Although children have little expectation of benefit themselves from vaccination, vaccinating children is necessary to secure benefits to overall well‐being that cannot otherwise be achieved. (It would also support challenge studies being performed [voluntarily] on low risk populations for a COVID‐19 vaccine, e.g. young people.)

It is often objected that utilitarianism leads to discrimination against those in ‘protected’ categories,^40^ such as the elderly, disabled, women, ethnic minority groups, etc.^41^ For example, in COVID‐19, it appears that elderly, male, obese, and BAME patients have a worse prognosis than other groups (to varying degrees). Utilitarians, it is argued, will give lower priority to some or all of these groups for access to limited resources and/ or a higher priority to isolating these groups, which is discrimination.

The first issue at hand is the accuracy of the information. For example, apparent differences in mortality between groups may be mere proxy correlations, that arise from unrelated factors such as faster spread amongst different groups in the community meaning there is uneven distribution of cases in the first place (we still do not know the true number of cases due to testing shortages in nearly all countries), the presence or absence of different groups in high‐risk occupations (in addition to uneven distribution of cases, there may be a ‘dose‐dependent’ effect of the viral load on the severity of illness making some workers more vulnerable), existing comorbidities that are correlated with different groups, but unrelated to them and should be considered separately, or poorer care due to bias or lack of access. Moreover, identification and analysis of these factors may lead to the ability to apply effective focussed measures such as equipping care homes with better testing and protective equipment, or focussed testing measures. Utilitarianism fails if it is applied unscientifically, without fine‐grained information, or if it fails to consider the best policy responses.

If the evidence associating a group of people with higher mortality is indeed both accurate and predictive of a higher mortality, and the association is of sufficient strength, and the proposed policy is both necessary and effective, then assigning resources or burdens such as lockdown selectively is no more discriminatory than other policies, such as the selective isolation of people on the basis of a proxy risk factor for infection, such as travel history or contact with someone who has COVID‐19 (this was the early strategy^42^).

Nevertheless, there would still be utilitarian reasons to reject policies that give lower priorities to these groups. In particular, these groups (with the exception of males) have already been disadvantaged, and indeed this disadvantage may even be the direct cause of vulnerability to COVID‐19. Justice requires that they not be further disadvantaged. Accepting the validity of justice need not mean rejecting utilitarianism. Utilitarians must consider all the effects of their policies and actions. If some policy will perpetuate or exacerbate discrimination or injustice with concomitant effects on well‐being, these must be considered. Loss of short‐term utility is justified by the larger long‐term gains of a more just society.

In any case, as we outlined at the beginning of this paper, utilitarianism is not necessarily a complete answer: one can sacrifice utility for other values. Thus, there might be straightforwardly utilitarian reasons for treating different groups in the same way: the resulting fractures in society arising from a policy that did not do so would ultimately cause a greater loss of well‐being. Or there might be pure justice reasons: upholding central values such as justice is more important than the net difference in expected health outcomes.

A key aspect of the law on discrimination is proportionality. In a pandemic, very large numbers of lives are at stake. Equality, even for those opposed to utilitarianism, is only one value amongst others. Discrimination may be proportionate if the stakes are high enough and alternative measures are not available.^43^

### 1.10 Separateness of persons

One prominent objection to utilitarianism is that it fails to respect the separateness of persons.^44^ One instantiation of this problem that is relevant to pandemic management is that utilitarianism can favour very small risk reductions spread over very large numbers of persons rather than the saving of one long life. Small goods can be summed to outweigh one large good.

Insofar as this is a problem, it can be avoided in practice by only comparing and summing comparable goods, for example lives. For example, one could count only the saving of lives or the saving of a life for a sufficiently long period of time (say 1 year) as a minimum good to be counted.

This vice can also be a virtue. The significant misery that a large number of people experience during lockdown (unemployment, depression, being victims of domestic violence, etc.) should not be ignored and must be recognized as an ethical cost. If that well‐being loss is great enough for a large enough number of people it could outweigh even the loss of some years of life for a relative few.

### 1.11 Conclusion

Utilitarianism is a demanding and counterintuitive theory. Why should we consider it? If the utilitarian course of action is not adopted, someone (often many) people will suffer or die avoidably. There may be good reasons (such as the preservation of liberty) to sacrifice well‐being or lives. But such choices need to be made transparently and in full awareness of their ethical cost. One must have good reasons to deliberately choose a course of action that will be worst overall.

Policy is often driven by politics or popular opinion, not ethics. This is morally wrong. Much of ethics in the public sphere involves social signalling, moralism and sometimes wishful thinking (for example, trying to wish away difficult ethical dilemmas). Careful consideration of the consequences of our actions requires us to face the facts and our values. A utilitarian approach is not simple, or easy. It requires that we choose the course of action that will benefit most people to the greatest degree, however difficult or counterintuitive that is.

There is some support for utilitarianism. In one survey investigating the public’s views on how to allocate intensive care beds amongst critically ill infants, we found the general public widely supported utilitarian allocations.^45^ They supported allocating the intensive care bed to save the infant with a greater chance of survival, who would have a longer life or less disability. They also supported saving the greater number. This suggests that there may be public support for the algorithm that we have proposed for the triage question. When people understand that there is an unavoidable need to choose between patients, they appear to recognize that securing the most benefit overall is both logical and ethical.

One of the psychological biases that dominates decision‐making is loss aversion. Losses loom larger than gains. And when we evaluate a policy we are liable to focus on the negatives, rather than the positives. Thus governments, such as East Asian governments, who radically curtail liberty and protect health and security are criticized for being overly authoritarian. Liberal governments that protect liberty and incur greater infection risks (such as the UK and Australia) are criticized for failing to protect the vulnerable and secure public health. There is no win the in the court of public opinion.

That is why we need, in the cool, calm hour, to set our policy objectives and priorities. Utilitarianism gives a clear framework for that. And it gives criteria to judge success.

The universal common ethical currency is well‐being. What matters to each of us is how well our lives go. This is the very heart and basis of utilitarianism: it takes an impartial approach to everyone’s well‐being. While people may argue other things matter (autonomy, privacy, dignity), everyone can agree that well‐being matters.

It is doubtful that any of the policies currently being adopted by any governments worldwide are purely or simply utilitarian. However, some are potentially reflecting more clearly and carefully about the costs and benefits of different courses of action and policy. The fundamental difficulty facing all of us during this pandemic is that we cannot know for certain which action will be best overall. We do not know what a utilitarian ‘archangel’ would choose: it would require a detailed understanding of the science and facts, the nature of well‐being and an exhaustive understanding of the consequences of our choices. But that is what we should be aspiring to. We must strive to get the facts straight on all the consequences of our choices. Our societies may then choose to embrace or choose not to embrace the utilitarian course. But at least we will then do so with a clear understanding of our values and the price we are willing to pay for them.

## Footnotes

^1^
Fink, S. (2020, March 28). U.S. Civil Rights Office rejects rationing medical care based on disability, age. *The New York Times*. Retrieved from https://www.nytimes.com/2020/03/28/us/coronavirus‐disabilities‐rationing‐ventilators‐triage.html

^2^
HHS Office for Civil Rights in Action. (2020, March 28). Bulletin: Civil rights, HIPAA, and the coronavirus disease 2019 (COVID‐19). Retrieved from https://www.hhs.gov/sites/default/files/ocr‐bulletin‐3‐28‐20.pdf

^3^
Smith, W. J. (2020, May 11). Bioethicist: Mandate COVID ‘contact tracing’ app, vaccinations. *National Review*. Retrieved from https://www.nationalreview.com/corner/bioethicist‐mandate‐covid‐contact‐tracing‐app‐vaccinations/

^4^
See Jackson, F. (1991). Decision‐theoretic consequentialism and the nearest and dearest objection. *Ethics, 101*, 461–482.

^5^
Hare, R. M. (1981). *Moral thinking: Its levels, method and point*. Oxford, UK: Clarendon Press.

^6^
Kahneman, D. (2011). *Thinking fast and slow*. London, UK: Farrar, Straus and Giroux.

^7^
Maani, M., & Galea, S. (2020, April 13). The true costs of the COVID‐19 pandemic. *Scientific American*. Retrieved from https://blogs.scientificamerican.com/observations/the‐true‐costs‐of‐the‐covid‐19‐pandemic/; Clemens, T., Popham, F., & Boyle, P. (2015). What is the effect of unemployment on all‐cause mortality? A cohort study using propensity score matching. *European Journal of Public Health, 25*(1), 115–121*;* Lundi, A., Falkstedt, D., Lundberg, I., & Hemmingsson, T. (2014). Unemployment and coronary heart disease among middle‐aged men in Sweden: 39 243 men followed for 8 years. *Occupational and Environmental Medicine, 71*, 183–188.

^8^
Savulescu, J., & Wilkinson, D. (2020, March 17). Who gets the ventilator in the coronavirus pandemic? These are the ethical approaches to allocating medical care. *ABC News Online*. Retrieved from https://www.abc.net.au/news/2020‐03‐18/ethics‐of‐medical‐care‐ventilator‐in‐the‐coronavirus‐pandemic/12063536

^46^
Data drawn from Ferguson, N. M., Laydon, D., Nedjati‐Gilani, G., Imai, N., Ainslie, K., Baguelin, M., … Ghani, A. C. (2020, March 16). Report 9: Impact of non‐pharmaceutical interventions (NPIs) to reduce COVID‐19 mortality and healthcare demand. https://doi.org/10.25561/77482. Retrieved from https://www.imperial.ac.uk/media/imperial‐college/medicine/sph/ide/gida‐fellowships/Imperial‐College‐COVID19‐NPI‐modelling‐16‐03‐2020.pdf

^9^
Triggle, N. (2020, March 21). Coronavirus deaths: What we don't know. *BBC News Online*. Retrieved from https://www.bbc.co.uk/news/health‐51979654

^10^
Hanlon, P., Chadwick, F., Shah, A., Wood, R., Minton, J., McCartney, G., … McAllister, D. A. (2020). COVID‐19 – exploring the implications of long‐term condition type and extent of multimorbidity on years of life lost: a modelling study [version 1; peer review: awaiting peer review]. *Wellcome Open Research 5*, 75.

^11^
Atherly, A. (2020, 24 March). Economic cost of flattening the curve. *The Incidental Economist*. Retrieved from https://theincidentaleconomist.com/wordpress/economic‐cost‐of‐flattening‐the‐curve/

^12^
Institute for Clinical and Economic Review. (2020, 31 January). 2020‐2023 value assessment framework. Retrieved from https://icer‐review.org/wp‐content/uploads/2019/05/ICER_2020_2023_VAF_013120‐4.pdf

^13^
Gates, B., & Chambers, R. (2015). From aspiration to action: What will it take to end malaria? Retrieved from http://endmalaria2040.org/assets/Aspiration‐to‐Action.pdf

^14^
BBC News. (2020, 30 March). Coronavirus: Trump signs into law largest bailout in US history. Retrieved from https://www.bbc.co.uk/news/world‐us‐canada‐52070718

^15^
Parfit, D. (1984). *Reasons and persons*. UK: Oxford University Press; Griffin, J. (1988). Well‐being: Its meaning, measurement and moral importance. Oxford, UK: Clarendon Press.

^16^
Wilkinson, D., & Savulescu, J. (2018). Prioritisation and parity: Which disabled infants should be candidates for scarce life‐saving treatment. In A. Cureton & D. Wasserman (Eds.), *Oxford handbook of philosophy and disability* (pp. 669–692). UK: Oxford University Press.

^17^
Arora, C., Savulescu, J., Maslen, H., Selgelid, M., & Wilkinson, D. (2016). The intensive care lifeboat: A survey of lay attitudes to rationing dilemmas in neonatal intensive care. *BMC Medical Ethics, 17*, 69.

^18^
Wilkinson, D., Brick, C., Kahane, G., & Savulescu, J. (2020). The relational threshold: A life that is valued, or a life of value? *Journal of Medical Ethics, 46*, 24–25.

^19^
Wilkinson, D., & Savulescu, J. (2014). A costly separation between withdrawing and withholding treatment in intensive care. *Bioethics, 28*(3), 127–137.

^20^
Glenza, J. (2020, 9 April). Who gets a ventilator? The 'gut‐wrenching' choices facing US health workers. *The Guardian*. Retrieved from https://www.theguardian.com/world/2020/apr/09/us‐healthcare‐workers‐make‐gut‐wrenching‐decisions‐coronavirus

^21^
Hancock, M. (2018, November 5). Prevention is better than cure – Matt Hancock’s speech to IANPHI. Retrieved from https://www.gov.uk/government/speeches/prevention‐is‐better‐than‐cure‐matt‐hancocks‐speech‐to‐ianphi

^22^
Friesen, P. (2018). Personal responsibility within health policy: Unethical and ineffective. *Journal of Medical Ethics, 44*, 53–58; Brown, R., & Savulescu, J. (2019). Responsibility in healthcare across time and agents. *Journal of Medical Ethics, 45*, 636–644.

^23^
Pillutla, V., Maslen, H., & Savulescu, J. (2018). Rationing elective surgery for smokers and obese patients: Responsibility or prognosis? *BMC Medical Ethics, 19,* 28.

^24^
Savulescu, J., & Persson, I. (2012). *Unfit for the future: The need for moral enhancement*. UK: Oxford University Press.

^25^
Jonsen, A. R. (1986). Bentham in a box: Technology assessment and health care allocation. *Law, Medicine and Health Care, 14*, 172–174.

^26^
Giles, C. (2020, May 12). Excess UK deaths in Covid‐19 pandemic top 50,000. *Financial Times*. Retrieved from https://www.ft.com/content/40fc8904‐febf‐4a66‐8d1c‐ea3e48bbc034

^27^
Worldometers. (2020, 6 May). *COVID‐19 Coronavirus Pandemic*. Retrieved from https://www.worldometers.info/coronavirus/

^28^
Savage, M. (2020, April 25). Coronavirus: Has Sweden got its science right? *BBC News Online*. Retrieved from https://www.bbc.co.uk/news/world‐europe‐52395866

^29^
Stewart, H., Boseley, S., Walker, P., & Elliott, L. (2020, March 16). PM tells Britons to avoid non‐essential travel and contact. *The Guardian*. Retrieved from https://www.theguardian.com/world/2020/mar/16/pm‐tells‐britons‐to‐avoid‐non‐essential‐contact‐with‐others

^30^
Wilkinson, D., & Savulescu, J. (2018). *Ethics, conflict and medical treatment for children: From disagreement to dissensus*. Elsevier.

^31^
The EuroMOMO hub. (2020). Graphs and maps. *EuroMOMO*. Retrieved from https://www.euromomo.eu, accessed May 5, 2020.

^32^
International Labour Organisation. (2020, March 18). Almost 25 million jobs could be lost worldwide as a result of COVID‐19, says ILO. Retrieved from https://www.ilo.org/global/about‐the‐ilo/newsroom/news/WCMS_738742/lang‐‐en/index.htm

^33^
Department of Health and Social Care. (2020, March 20). Crucial medicines protected for coronavirus (COVID‐19) patients. Retrieved from https://www.gov.uk/government/news/crucial‐medicines‐protected‐for‐coronavirus‐COVID‐19‐patients

^34^
Forero, J., & Pérez, S. (2020, April 23). Coronavirus pandemic prompts race in Latin America to build cheaper ventilators. *Wall Street Journal*. Retrieved from https://www.wsj.com/articles/coronavirus‐pandemic‐prompts‐race‐in‐latin‐america‐to‐build‐cheaper‐ventilators‐11587634202

^35^
Bui, T. (2020, May 1). Aggressive testing and pop songs: How Vietnam contained the coronavirus. *The Guardian*. Retrieved from https://www.theguardian.com/commentisfree/2020/may/01/testing‐vietnam‐contained‐coronavirus

^36^
Broadbent, A., & Smart, B. J. H. (2020, March 23). Why a one‐size‐fits‐all approach to COVID‐19 could have lethal consequences. *The Conversation*. Retrieved from https://theconversation.com/why‐a‐one‐size‐fits‐all‐approach‐to‐covid‐19‐could‐have‐lethal‐consequences‐134252?utm_medium=email&utm_campaign=Latest%20from%20The%20Conversation%20for%20March%2024%202020%20‐%201572415047&utm_content=Latest%20from%20The%20Conversation%20for%20March%2024%202020%20‐%201572415047+CID_5c42b122e00729810ed80b4b87906ccc&utm_source=campaign_monitor_global&utm_term=argue

^37^
Vaswani, K. (2020, 19 March). Coronavirus: The detectives racing to contain the virus in Singapore. *BBC News Online*. Retrieved from https://www.bbc.co.uk/news/world‐asia‐51866102

^38^
Valentino‐DeVries, J. (2020, March 19). Translating a surveillance tool into a virus tracker for democracies. *The New York Times*. Retrieved from https://www.nytimes.com/2020/03/19/us/coronavirus‐location‐tracking.html?referringSource=articleShare

^39^
Bambery, B., Douglas, T., Selgelid, M., Maslen, H., Giubilini, A., Pollard, A., & Savulescu, J. (2018). Influenza vaccination strategies should target children. *Public Health Ethics, 11*(2), 221–234.

^40^
Savulescu, J., Cameron, J., & Wilkinson, D. (2020, in press). Equality or utility? Ethics and law of rationing ventilators. *British Journal of Anaesthesia*. https://doi.org/10.1016/j.bja.2020.04.011

^41^
Harris, J. (1987). QALYfying the value of life. *Journal of Medical Ethics, 3*, 1–18; Harris, J. (1995). Double jeopardy and the veil of ignorance – A reply. *Journal of Medical Ethics, 21*, 151–157.

^42^
Cameron, J., & Savulescu, J. (2020). Why lock down of the elderly is not ageist and why levelling down equality is wrong. Under review.

^43^
Savulescu et al., op. cit. note 40.

^44^
Rawls, J. (1971). *A theory of justice*. Cambridge, MA: Belknap Press of Harvard University Press.

^45^
Arora et al., op. cit. note 17.

